# Supplementary material for: The global burden of age-related hearing loss among individuals aged 60 years and older: An analysis for the global burden of disease study 2021 and predictions to 2050
Source: PLoS One. 2026 Jan 16;21(1):e0340498. doi: 10.1371/journal.pone.0340498 (PMC12810803; doi:10.1371/journal.pone.0340498)
Supplement: Attachment — Submitted filename: PONE-D-25-09817_rev.docx [file pone.0340498.s003.docx]

The manuscript by Li et al. presents well written analysis of the global burden of age-related hearing loss (ARHL) from 1990 to 2021, with projections extending to 2050. Based on data from the GBD study, the authors provide evidence that ARHL and the associated burden are increasing globally, with significant differences among regions and development status. The finding are consistent with the information and projections from the World Health Organization and contribute valuable insights for global hearing health in people ≥ 60 years.

Overall, methodology is clear and could be replicated.

**Comments**

- Ln 47-48: reference for this line (7) is about employment not about cognitive decline. Also, the idea “…accelerates cognitive decline” could be interpreted as an overstatement.
- The information contained in Table 1 could be divided into two tables for clearer presentation (Prevalence and YLD).
- Ln 83: may be useful for the readers to have de detail of the direction of change in EAPC (eg. greater than 0 = increase).
- Figure 2: It may be helpful to choose an alternative color palette, considering the challenges that red–green combinations pose for individuals with color vision deficiency. A useful reference could be the following work: Guo, Z., Ji, W., Song, P. et al. Global, regional, and national burden of hearing loss in children and adolescents, 1990–2021: a systematic analysis from the Global Burden of Disease Study 2021. BMC Public Health 24, 2521 (2024). https://doi.org/10.1186/s12889-024-20010-0
- Figure 6 shows the frontier analysis of SDI and YLDs of ARHL among individuals aged 60 and older of the 1990-2021 period. The caption of the figure mention “Frontier analysis…. in 2021”. Please check and modify.

A plot B could be added to the figure to show 2021 data, highlighting some countries with greater potential to reduce ARHL. For the dots representing individual countries, it may be useful to use different colors for increase or decrease in the effective difference. This article shows something similar: Chen, X., Zhou, C. W., Fu, Y. Y., Li, Y. Z., Chen, L., Zhang, Q. W., & Chen, Y. F. (2023). Global, regional, and national burden of chronic respiratory diseases and associated risk factors, 1990-2019: Results from the Global Burden of Disease Study 2019. *Frontiers in medicine*, *10*, 1066804. https://doi-org.uchile.idm.oclc.org/10.3389/fmed.2023.1066804

- The focus of this work was people 60 and older, which is described as a limitation of the study by the authors (not including younger population). However, I do not consider this a major limitation, as this age group is relevant for the analysis of ARHL.

However, if this point is important for the authors, it would be helpful to support the choice of age cutoff with data from WHO and other sources to strengthen the rationale. For example, including individuals aged 50 and older.
