# Supplementary material for: The global burden of age-related hearing loss among individuals aged 60 years and older: An analysis for the global burden of disease study 2021 and predictions to 2050
Source: PLoS One. 2026 Jan 16;21(1):e0340498. doi: 10.1371/journal.pone.0340498 (PMC12810803; doi:10.1371/journal.pone.0340498)
Supplement: Attachment — Submitted filename: Response to Reviewers.docx [file pone.0340498.s005.docx]

**Response to Reviewers**

Dear Editor,

We sincerely thank you and the reviewers for your time and valuable comments on our manuscript. We have carefully considered all suggestions and revised the manuscript accordingly. We believe these revisions have significantly improved the quality of our work. All changes have been highlighted in tracked changes.

**Editor's Comments**

We greatly appreciate the editor's careful review and helpful guidance.

**Comment 1: Please ensure that your manuscript meets PLOS ONE's style requirements, including those for file naming.**

Response: Thank you for this reminder. We have carefully reviewed and ensured full compliance with PLOS ONE's style requirements.

**Comment 2: When you resubmit, please ensure that you provide the correct grant numbers for the awards you received for your study in the 'Funding Information' section.**

Response: We note the discrepancy mentioned between the 'Funding Information' and 'Financial Disclosure' sections. During the original submission, we could only locate the 'Funding Information' section in the submission interface and entered all funding details there. To ensure consistency, our corrected funding statement is: **This work is supported by the National Natural Science Foundation of China (82301789), the Program of Excellent Doctoral (Postdoctoral) of Zhongnan Hospital of Wuhan University (Grant No. ZNYB2021020), Youth Interdisciplinary Special Fund of Zhongnan Hospital of Wuhan University (Grant No. ZNQNJC2023007).**

**Comment 3: Your ethics statement should only appear in the Methods section of your manuscript. If your ethics statement is written in any section besides the Methods, please move it to the Methods section and delete it from any other section.**

Response: Thank you for your clarification. The ethics statement has been moved exclusively to the Methods section as requested.

**Comment 4: We note that Figures 2 and S2 in your submission contain map images which may be copyrighted. All PLOS content is published under the Creative Commons Attribution License (CC BY 4.0)... For these reasons, we cannot publish previously copyrighted maps or satellite images created using proprietary data, such as Google software (Google Maps, Street View, and Earth).**

Response: We appreciate your careful attention to copyright compliance. We have removed Figures 2 and S2 to avoid any potential copyright issues.

**Reviewer #1**

We sincerely thank Reviewer #1 for the thorough and constructive review. We particularly appreciate you bringing to our attention the article "Global trends and burden of age-related hearing loss: A 32-Year Study" (Archives of Gerontology and Geriatrics, 2025, https://doi.org/10.1016/j.archger.2025.105847), which was published during our submission period. We have now addressed how our work differs from and complements this important publication.

**Comment 1 - Introduction: A more detailed description of the Global Burden of Disease (GBD) initiative would be helpful, including its purpose, scope, and the mechanisms by which data are collected, standardised, and modelled. With regard to age-related hearing loss (ARHL) specifically, I recommend providing a brief explanation of how the estimates are generated, including the sources used (e.g. national health surveys, clinical records, scientific literature) and the operational definitions of hearing loss employed.**

Response: We thank the reviewer for this valuable suggestion. We have added detailed GBD methodology and ARHL estimation procedures in the Introduction and Methods sections, which we believe substantially strengthens the manuscript.

**Comment 2 - Methods: While the manuscript adequately describes the methods used, I recommend explicitly stating which statistical software or packages were employed for each type of analysis (e.g. linear regression, Bayesian models and inequality analysis).**

Response: We agree that transparency is crucial for reproducibility. We have now added comprehensive statistical software and R package information in the Methods section.

**Comment 3 - Discussion: It would be beneficial to strengthen the connection between your findings and their potential implications for public policy and health planning, especially considering regional disparities according to development levels. Additionally, I suggest explicitly referencing the findings from the previously cited published study, and elaborating further on how your results complement, nuance, or challenge those findings. You may wish to briefly discuss the limitations of relying exclusively on modeled secondary data, as is the case with GBD, and the potential implications this has for the precision of regional estimates—particularly in countries with limited availability of primary data.**

Response: We greatly appreciate these insightful suggestions. The Discussion section has been substantially enhanced with:

- Strengthened policy implications and regional disparities analysis
- Explicit comparison with the 2025 recently published study
- Critical discussion of GBD modeled data limitations These additions have significantly improved the depth and practical value of our manuscript.

**Reviewer #2**

We are grateful to Reviewer #2 for the detailed and constructive feedback that has helped improve the clarity and accessibility of our manuscript.

**Comment 1: Ln 47-48: reference for this line (7) is about employment not about cognitive decline. Also, the idea "...accelerates cognitive decline" could be interpreted as an overstatement.**

Response: Thank you for catching this error. We have corrected the reference and modified the wording from "accelerates cognitive decline" to "is associated with increased risk of cognitive decline" for better accuracy.

**Comment 2: The information contained in Table 1 could be divided into two tables for clearer presentation (Prevalence and YLD).**

Response: We agree that this improves clarity. We have divided the content into Table 1 (Prevalence) and Table 2 (YLD). Thank you for this helpful suggestion.

**Comment 3: Ln 83: may be useful for the readers to have the detail of the direction of change in EAPC (eg. greater than 0 = increase).**

Response: Excellent point. We have added clear EAPC interpretation in the Methods section for reader clarity.

**Comment 4: Figure 2: It may be helpful to choose an alternative color palette, considering the challenges that red–green combinations pose for individuals with color vision deficiency.**

Response: We appreciate your attention to accessibility. As noted in our response to the Editor, Figure 2 and S2 has been removed due to copyright concerns.

**Comment 5: A plot B could be added to the figure to show 2021 data, highlighting some countries with greater potential to reduce ARHL. For the dots representing individual countries, it may be useful to use different colors for increase or decrease in the effective difference.**

Response: Thank you for this excellent suggestion. We have enhanced Figure 6 with Panel B showing 2021 data with differential coloring as recommended. Corresponding descriptive text has been added to the Results section.

**Comment 6: The focus of this work was people 60 and older, which is described as a limitation of the study by the authors (not including younger population). However, I do not consider this a major limitation, as this age group is relevant for the analysis of ARHL.**

Response: We appreciate your perspective on this point. Based on your feedback, we have removed this from the limitations section, as we agree that the 60+ age group focus is indeed appropriate for ARHL analysis.

Once again, we sincerely thank the editor and reviewers for their time and effort in reviewing our manuscript. We believe that the manuscript has been substantially improved through this revision process. We hope that you will find the revised version suitable for publication in *PLoS One*.

Sincerely,

Ling Li

Corresponding author: Ling Li, MD, PhD

Department of Nuclear Medicine, Zhongnan Hospital of Wuhan University, No.169 Donghu Road, Wuhan, 430071, China

E-mail address: linglizn21@whu.edu.cn
